# SARS-COV-2 vaccine: first-month results of a six-month follow-up study

**DOI:** 10.3906/sag-2106-63

**Published:** 2021-10-17

**Authors:** Burak METE, Ferdi TANIR, Hakan DEMİRHİNDİ, Ertan KARA, Filiz KİBAR, Salih ÇETİNER, Aslıhan CANDEVİR, Rabia DAL

**Affiliations:** 1 Department of Public Health, Faculty of Medicine, Çukurova University, Adana, Turkey; 2 Department of Medical Microbiology and Çukurova University Balcalı Hospital Central Laboratory, Faculty of Medicine, Çukurova University, Adana, Turkey; 3 Department of Immunology and Çukurova University Balcalı Hospital Central Laboratory, Faculty of Medicine, Çukurova University, Adana, Turkey; 4 Department of Infectious Diseases and Clinical Microbiology, Faculty of Medicine, Çukurova University, Adana, Turkey

**Keywords:** SARS-COV-2, vaccine, adverse events, immunisation, seroconversion

## Abstract

**Background/aim:**

Various COVID-19 vaccines are being developed around the world. Important questions to be answered regarding vaccines are efficacy, safety, and whether antibodies are protective when used in different communities. This study aimed to determine seroconversion rates of the inactivated SARS-CoV-2 vaccine in healthcare workers in a hospital and short-term adverse events due to the vaccine.

**Materials and methods:**

The study carried out in Çukurova University, Turkey, comprised of 282 healthcare workers who received two doses of the inactivated SARS-CoV-2 vaccine administered in two 3 μg doses, 28 days apart. On day 28, after the second dose, anti-S-RBD IgG and total anti-spike and anti-nucleocapsid IgM and IgG antibodies against SARS-CoV-2 were detected by using in vitro chemiluminescence immunoassay method.

**Results:**

The mean age of participants was 39.06±10.65 (min=21, max=65) with 43.6% males and 56.4% females. On day 28, after the second dose, the seroconversion rates were found to be 92.9% for total anti-spike and anti-nucleocapsid IgG and 15.2% for IgM and 98.2% for anti-S-RBD IgG antibodies and having natural COVID-19 prior to vaccination, age and comorbidity were found to be significant factors for immunogenicity. The incidence of at least one adverse event was found as 29.8% after the first dose and 24.1% after the second dose, with the most common adverse events of having pain at the injection site, weakness, fatigue, and headache.

**Conclusion:**

On day 28, after the second dose of 3 μg of the inactivated SARS-CoV-2 vaccine administration, a high rate of seroconversion was observed with no serious adverse event. Natural COVID-19 history, age, and comorbidity were significant contributors to the formation of a strong immune response. It can be concluded that a third dose may be considered in people aged 50 years and older and those with comorbidities.

## 1. Introduction

The COVID-19 pandemic, caused by severe acute respiratory syndrome-coronavirus 2 (SARS-CoV-2) belonging to the genus Betacoronavirus of the family Coronaviridae, has caused almost 153 million infections and more than 3.2 million deaths worldwide as of May 6, 2021.^1^ The transmission rate is higher in SARS-CoV-2 in comparison to other species of coronavirus [1]. Various COVID-19 vaccines are in development around the world, aiming to stimulate a primary immune response leading to the development of memory B and T cells against the SARS-CoV-2 virus and, therefore, a protection against subsequent COVID-19 infections. S protein of the virus plays an essential role in viral binding (S1 subunit), fusion (S2), entry and transmission, and elicits highly potent neutralizing antibodies (nAbs). The receptor-binding domain (RBD) of S1 directly interacts with host receptors (i.e. human-angiotensin-converting-enzyme-2) and is the most critical target for nAbs, which interrupts this interaction. Thus, anti-SARS-CoV-2 S-RBD IgG antibody level in human serum or plasma correlates with protective immune responses in individuals recovered from SARS-CoV-2 infection and also reflects herd immunity at the population level, informing clinical management of patients with past or ongoing infection.^2^

Vaccines are considered the primary method for preventing viral infections and for containing epidemics, therefore, a vital tool in reducing mortality in a pandemic. They are categorised according to their compositions as adenovirus vector, mRNA, DNA, live attenuated, inactivated, or subunit type vaccines [2]. Inactivated vaccines incorporate viruses inactivated by using heat, chemicals, or radiation, and they do not cause any infection; hence, they are considered to be safe for immunocompromised individuals. They may have some logistical benefits like storage at room temperature. One disadvantage is that they frequently require multiple doses (boosters) as they do not create a cell-mediated response [3]. They can have added adjuvants to spare doses and increase the available quantity of the vaccines [4]. The inactivated SARS-CoV-2 vaccine (Vero Cell) has been shown to be safe and able to induce SARS-CoV-2 specific nAbs in mice, rats, and nonhuman primates [5]. In phase 2 clinical trials, this vaccine was observed to produce good immunogenicity and a low incidence of adverse events [6]. But it is still not known if formed antibodies are protective (surrogate) in wider public use.^3^ This is particularly important when we consider that none of the vaccines in use has completed phase trials, with especially phase 4 results completely missing, as they have been introduced for public use following Emergency Use Listing evaluation.

This study aimed to determine seroconversion rates and levels of the neutralising and non-neutralising antibodies, the effect of individual characteristics including comorbidities or history of natural COVID-19 after the second dose of the inactivated SARS-CoV-2 vaccine among healthcare workers in a hospital and short-term adverse events due to the vaccine in order to help decision-makers by comparing doses, schedules, and the need for additional doses.

## 2. Materials and methods

### 2.1. Study design and participants

This is a prospective cohort study carried out at Çukurova University in March 2021 and included health workers, who had been vaccinated with two doses of the inactivated SARS-CoV-2 vaccine in the context of public vaccination program by the Turkish Ministry of Health, with health workers constituting the first line of the vaccination target population. The permissions for the study were obtained from the Scientific Research Platform of the Turkish Republic Ministry of Health, Balcalı Hospital Managerial Board, and the Scientific Ethics Committee of Faculty of Medicine, Çukurova University (Decision no:108). The incidence of adverse events in the vaccine cohort was determined to be 19% after the administration of 3 μg (corresponding to 600 SU) of the inactivated SARS-CoV-2 vaccine during Phase 2 clinical trials [6]. The minimum sample size was calculated as 220 participants by assuming type-1 error as 0.05 and type-2 error as 0.20 for the determined adverse events rate. In order to increase the power of the study, as many participants were included in the study as the financial budget of the project allowed, a total of 282 participants were reached out. The proportional distribution of the profession subgroups in the sample (i.e. study group) reflected their distribution in the study universe. The total number of staff working in the hospital is 4.285, with 1052 doctors (24.6%), 1033 nurses(24.1%), and 2200 (51.3%) other groups consisting of emergency medical technician, biologist, environmental engineer, pharmacist, electrician, EMG technician, security staff, nursing staff, occupational health and safety expert, chemist, laborant, officer, audiologist, cleaning staff, psychologist, radiology technician, secretary, driver, and mechanist. The participants reported their working departments under the as following names: emergency, emergency patient admission office, COVID-19 sampling unit, forensic case office, family medicine, operating room, anatomy, vaccination unit, occupational health and safety unit, neurosurgery service, IT department, biophysics department, biostatistics department, biomedical unit, paediatrics service, newborn service, COVID-19 polyclinic, COVID intensive care unit, COVID service, paediatric surgery department, paediatric emergency department, paediatric service, internal medicine department, oncology department, dean’s office, archive, tissue typing laboratory, dialysis unit, pharmacy services, pharmacy, electricity control unit, EMG unit, endoscopy unit, infectious diseases polyclinic, infectious diseases service, physical therapy and rehabilitation, gastroenterology service, general surgery service, pneumology polyclinic, pulmonology service, pulmonary function tests unit, radiology imaging unit, eye-bank, ophthalmology service, daily patient unit, security, patient admission, public relations, hospital chief directorate, hospital heating centre, haemapheresis unit, haematology laboratory, haematology service, haematology polyclinic, haemodialysis unit, nursing services directorate, nursing services department, histology-embryology, nephrology, human resources, obstetrics, in vitro fertilization centre, quality control directorate, cardiology department, ear-nose-throat department, delivery room, acclimatisation centre, financial affairs, central laboratory, PCR unit, central sterilization unit, directorate, nephrology, neurology service, neurology EEG unit, neurology intensive care, nuclear medicine, oncology, pathology, paediatric, plastic surgery, psychiatry, radiation oncology, radiology, radiology NMR unit, radiology secretariat, health board, sterilization, surveillance, drivers’ office, trusteeship department, technical coordinator, medical biochemistry, medical genetics, deanship, faculty of medicine multidisciplinary laboratory, urology, chief clerkship and neonatal intensive care unit.

The participants were randomly selected from a list of 3000 vaccinated health workers. Two substitution lists were prepared to eliminate the possibility of missing any participant and to preserve the pre-study randomisation. Nobody in the main (first) list refused to participate in the study; therefore, there has not been a need to use the substitution lists. Blood samples were taken after the participants had been informed about the study and had signed an informed consent form. Blood was taken from the participants included in the study by the experienced and educated nurses already working at the blood collection unit of the central laboratory of the hospital. Since the people included in the study were both the personnel working in our hospital and among the first individuals that have been vaccinated, the vaccination dates were obtained from the personnel vaccination lists. Both the vaccination list and the appointment dates of the individuals on the e-government application were considered in determining the 28th day after the second dose. The appointments were made after a large proportion of the health workers in the hospital were vaccinated, giving rise to the list of 3000 vaccinated personnel. The people selected to be included in the study were identified immediately after the second dose of the vaccines, and all of them were contacted on their mobile phones, and their appointments were confirmed after they had agreed to volunteer for the study. The blood sample collection date for each individual was given 28 days after the second dose of vaccination. Reminder text messages were sent to people two days before the planned time of blood collection, and people who did not respond were recalled and reminded. The participants answered a questionnaire form about sociodemographic characteristics, history of a natural COVID-19 disease, comorbidities like cardiovascular, endocrinological, immunological, pulmonary and other chronic diseases, vaccination, and adverse events.

### 2.2. Vaccine information

The generic name of the vaccine administered to health workers by the Turkish Ministry of Health is “inactivated SARS-CoV-2 vaccine (Vero Cell) (CoronaVacTM)”, with aluminium hydroxide developed by Sinovac Biotech Ltd., Life Sciences Lab., China. The vaccine was administered intramuscularly in the deltoid region of the upper arm with a dosage of 3 μg/0.5 ml. The two doses were administered 28 days apart. Vaccines were transferred, stored at 2–8°C, and administered following cold chain principles already in use by and, therefore, familiar to health institutions performing vaccinations.

### 2.3. Immunogenicity assessments

The project aimed to measure SARS-CoV-2 S-RBD immunoglobulin G (IgG), total anti-spike and anti-nucleocapsid immunoglobulin M (IgM) and IgG antibody concentrations in the same individuals repetitively in the first, third, and sixth months. This first step of the project aimed to reveal anti-SARS-CoV-2-S-RBD-IgG, total anti-spike/anti-nucleocapsid IgM and IgG concentrations at the end of the first month after the administration of the second dose of the inactivated vaccine. The second and third steps of the project are planned to be performed at the third and sixth months after the second dose, respectively. Anti-SARS-CoV-2-S-RBD-IgG and total anti-spike/anti-nucleocapsid-IgG antibodies were to be measured in the same participants. About 5 mL of blood samples were collected into biochemistry tubes with vacuum gel. The sera were extracted by centrifugation at 3000 × g for 10 min and kept at 2–8°C for 1–3 days. Test calibrators and controls were worked first. After the control results were observed to be within the expected ranges, the samples were tested by trained experts in the accredited (by the Joint Commission International (JCI) since 2006) Central Laboratory of Çukurova University.

The blood samples stored at 2–8°C were brought to room temperature on the working day and examined collectively after all participants were sampled. The participants were not informed about their antibody concentration at the first month (in a single-blind format) to avoid a possible effect on clinical complaints or outcomes as they would be followed repetitively for 6 months.

The test method used for the qualitative detection and differentiation of IgM (capture method) and IgG antibodies (indirect method) to SARS-CoV-2 in human serum and serum in separating gel tubes is a chemiluminescence reaction based on the measurement of light signals by a photomultiplier as relative light units (RLUs) proportional to the concentration of antibodies present in the sample.^4^,^5^ The test is only for use according to the Food and Drug Administration’s Emergency Use Authorization.^6^ The SARS-CoV-2 S-RBD IgG test is also an indirect CLIA and has a high correlation with VNT50 titres (R=0.712), where VNT stands for “Virus Neutralization Test”, which is a gold standard for quantifying the titre of nAbs for a virus [7]. The results are expressed in absorbance units (AU/mL) and reported to the end-user as “reactive (i.e. a result ≥1.00 AU/mL)” or “non-Reactive (i.e. a result <1.00 AU/mL)”.^7^ The validity of the tests is summarised in Table 1.^8^

### 2.4. Statistical analyses

Data were analysed by SPSS 22 statistical analyses package (2013, IBM, New York, U.S.A). Kolmogorov–Smirnov test was used to test the normality for continuous variables. Mann–Whitney U and Kruskal–Wallis tests were used for not normally distributed variables. Fisher’s, Yates, or Pearson chi-square tests were used for categorical variables. A value of p < 0.05 was considered significant.

## 3. Results

The mean age of 282 participants included in the study was 39.06±10.65 (min–max = 21–65). Among them, 16% reported a natural COVID-19 history before getting vaccinated. The mean time interval between the disease and the vaccination was calculated as 111.27±77.97 days (min–max = 29–330). The sociodemographic characteristics and any natural COVID-19 history of the participants are given in Table 2.

**Figure 1:**
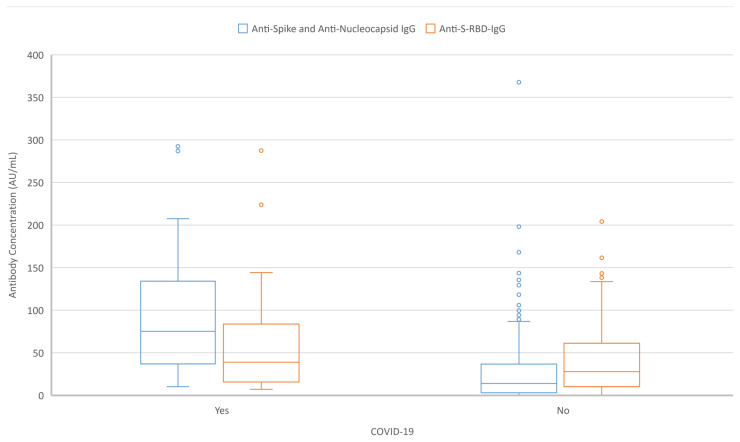
Anti-S RBD IgG (AU/mL) and total anti-spike and anti-nucleocapsid IgG (AU/mL) concentrations according to the history of COVID-19 prior to vaccination ^*^Yes: The participant had a history of natural COVID-19 prior to vaccination ^*^No: The participant did not have a history of natural COVID-19 prior to vaccination

At the first month after the administration of the second dose of the inactivated vaccine, 277(98.2%) participants were found to be reactive for anti-S-RBD-IgG antibodies, 262(92.9%) for total anti-spike/anti-nucleocapsid-IgG antibodies and 43(15.2%) for total anti-spike/anti-nucleocapsid-IgM antibodies. When seroconversion rates were examined according to age, sex, presence of chronic diseases, and having natural COVID-19 prior to vaccination, only participants with a natural history of COVID-19 presented significantly higher seroconversion rates for total anti-spike/anti-nucleocapsid-IgG (p = 0.027). All individuals with non-reactive IgG concentrations for all types of antibodies did not report a prior natural COVID-19 (Table 3) (Figure 1).

**Figure 2:**
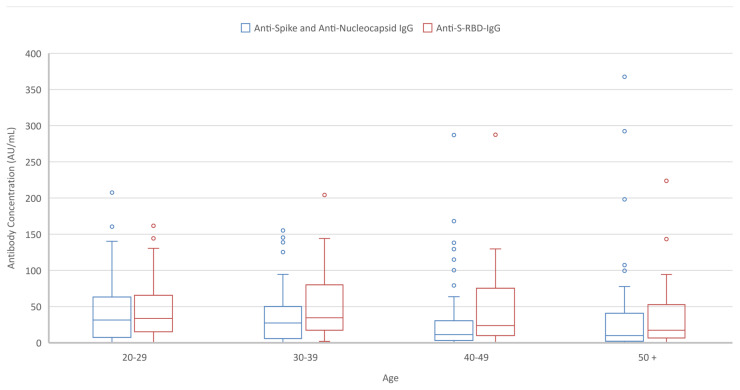
Anti-S RBD IgG (AU/mL) and total anti-spike and anti-nucleocapsid IgG (AU/mL) concentrations according to the age groups (including all participants)

Significantly higher antibody titrations were observed in participants with a pre-vaccination history of natural COVID-19, but the time interval between the disease and the vaccination was found not to significantly alter antibody concentrations. Total anti-spike/anti-nucleocapsid-IgG titrations of participants aged 20–29 years were found to be significantly higher compared to those in the 40–49 and 50 and older age groups. Anti-S-RBD-IgG titrations were significantly higher in the 30–39 years age group compared to 50 and older age groups. Additionally, anti-S-RBD-IgG titrations were found to be higher in individuals with chronic diseases (Table 4) (Figure 2).

No COVID-19 cases were observed to occur in the vaccine cohort between the two doses and until the end of the first month after the second dose of vaccination. In the vaccine cohort, the incidence of at least one adverse event was found to be 29.8% between the two doses and 24.1% after the second dose with the most frequently observed ones in both periods as pain at the injection site, weakness, fatigue, and headache. Some participants declared to have experienced more than one side effect. After the first dose, multiple adverse events were observed in 46(16.3%) participants. Concurrent adverse events were reported as “Numbness in the vaccinated arm + Fatigue + Weakness” and “Headache + Dizziness”. After the second dose, multiple adverse events frequency regressed to 13.4% (38 people), with the most frequent concurrent one being reported as “weakness fatigue” (Table 5).

## 4. Discussion

In the COVID-19 pandemic, the recent introduction of vaccines has strengthened our hand in combating the epidemic. As of May 6, 2021, among developed vaccines, 96 are reported to be at clinical and 184 at preclinical stage.^9^ But vaccines have given rise to the discussion of new problems like how to vaccinate the whole society, inequalities in the supply of vaccines, weaknesses in the protection by vaccines, and resistance to get vaccinated. It seems likely that ensuring herd immunity will be still in action for some more time. Kuldorff et al. stated that a focused protection approach would ensure herd immunity after 3 to 6 months and then unprotected individuals could return to normal life. Walensky stated that the herd immunity point was not determined and that it was not clear how stable this immunity would be. Most experts believe this to happen in the second half of 2021 at the earliest, and debate over what to do in the meantime seems likely to continue. Perhaps the most important of these discussions is the effectiveness and safety of the vaccines in use and how long the vaccine-induced immunity will continue and what the protection rates from the clinical disease are. Outbreak patterns are supposed to be related to the duration of immunity [8]. Previous studies about other coronavirus types demonstrated that annual or biennial patterns could be observed if the immunity lasted 40 weeks or two years, respectively, not ignoring the influence of cross-immunity.^10^ On January 13, 2021, the inactive vaccine “CoronaVac, in suspension form of 600 SU/0.5 mL for IM injection” was quickly granted the Emergency Use Approval by the Turkish Ministry of Health, aiming to reduce the spread of COVID-19 in the community. This vaccine is currently being implemented in 24 countries and is being evaluated also in seven phase 3 trials.^11^ As of August 27, 2021, a total of 91.74 million doses were administered in Turkey, with 47.24 million of the first dose and 36.14 million of the second.^12^

Our study aimed to determine the efficacy of this inactivated vaccine administered in the context of this program of the Turkish Ministry of Health. It is worth reminding that the administrations were not part of any phase trials or interventional studies. In our study, at day-28 after the second dose, among participants who received two doses of the inactivated vaccine (as 3 μg/dose), the seroconversion rate was found to be 15.2% for total anti-spike/anti-nucleocapsid-IgM, 92.9% for total anti-spike/anti-nucleocapsid-IgG and 98.2% for anti-S-RBD-IgG antibodies. The incidence of adverse events was found as 29.8% between the two doses and 24.1% at day-28 following the second dose, the most prominent adverse event being pain at the injection site (16.0% between two doses of the vaccine, and 15.6% at day-28 after the second dose), followed by weakness (10.6% versus 15.6%) and fatigue (9.9% versus 10.3%) (Table.5). These correlated with the package insert of the vaccine, based on previous clinical studies, and classified among “the very common (>%10) adverse reactions” like injection site pain, headache, and fatigue.

Zhang et al. [6] performed two-phase trials (1/2) for the same inactivated vaccine comparing 0–14 and 0–28-days regimes and two doses (3 μg vs 6 μg). Both trials supported our findings as better seroconversion specific to nAbs was observed in 0–28-days cohorts. Similarly, the seroconversion was higher in the 3μg dose group of phase-1, but in the 6 μg dose group of phase-2, although the difference was very small (3%). Again, adverse events rates were also similar, being lower in 3 μg dose and 0–28-day cohort in both trials, supporting our findings (Table 5).

Xia et al. [9] performed also two-phase trials for the same inactivated vaccine. They compared three different doses (2.5, 5, and 10 μg) as a 0–28–56-days regime in phase-1. If we consider 2.5 μg dose as the closest one to our 3-μg dose, the seroconversion specific to nAbs was found as 100%. In phase-2 they compared the same dose (5 μg) between 0–14 and 0–21-days regimes. Specific IgG binding antibody response was better in the 0–21-day regime (100%) compared to the 0–14-day regime (85.7%), while seroconversion specific to nAbs was equal in both regimes. When they compared geometric mean titres (GMT) of nAbs and specific IgG-binding antibody responses, they found both GMTs to be the highest in the 2.5 μg dose group in phase-1 and in 0–21-day regime in phase-2, supporting our findings. When Xia et al. examined adverse events rates, the safest regime was reported in the 5-μg group of the 0–14-day cohort. This is different from our findings, but it is noteworthy to indicate that Xia et al reported adverse events on day 7, which seems too early compared to our results of 28 days.

Wu et al. [10] conducted a phase-1/2 trial, which followed a 0–28-day regime, targeting healthy adults 60 years old and older (mean age 66.6±4.7), and found similar results with our study regarding age profiles; a 100% of seroconversion specific to nAbs was observed in 3 μg dose of phase-1 and 98.0% in phase-2. Our study findings also supported the effectiveness of this dosage and schedule. In our study, the seroconversion rate in the 50 and older age group was found as 89.1%, with a decreasing rate with increasing age (95.7% in 20–29 years of age group). This emphasizes the need for advanced phase studies in order to clarify the immunogenicity of the inactivated vaccine in terms of age groups and the post-vaccination period. A comprehensive discussion comparing the results of previous research/phase trials has been added as Supplement 2. In a phase 3 trial about the inactivated CoronaVac performed in Turkey by Tanrıöver et al. [11], involving 1413 participants (981 in the vaccine group and 432 in the placebo group), the immunogenicity analyses revealed a seropositive for RBD-specific total antibody of 89.7% in vaccine recipients and 4.4% in placebo recipients, with the seropositivity decreasing with increasing age. In another study from Turkey by Bayram et al. [12] the seropositivity for anti-spike antibodies was detected in 99.6% of healthcare workers, antibody titres being higher in those with COVID-19 history before vaccination. Antibody positivity and median antibody titres were significantly lower in healthcare workers with chronic diseases.

The results of the trials mentioned are consistent with our findings as the dose in Turkey was almost the same (3 μg). But considering the finding that the 3-dose regimens proved the best option has led us to hypothesise that an additional dose could be beneficial in achieving both the seroconversion and higher antibody concentrations. Our hypothesis is based on our finding that all participants (100.0%) with a history of natural COVID-19 were reactive for anti-S-RBD, total anti-spike and anti-nucleocapsid IgG antibodies (p = 0.027) and antibody concentrations were significantly higher with a median value of 38.99 (7.18–287.5) AU/mL for anti-S-RBD (p = 0.033) and 75.27 (10.16–292.40) AU/mL for the total anti-spike and anti-nucleocapsid IgG antibodies (p < 0.001). The details of all referred trails are given in the Supplemental Table.

Differently from phase trials, our study did not include solely sensitive and healthy participants; but individuals who had previous natural COVID-19 history or accompanying chronic diseases were also included in the study. Our results demonstrated that the seroconversion rate and antibody responses were higher in individuals who had a history of natural COVID-19. When the immunogenicity was evaluated based on individual characteristics; sex was not found to significantly alter seroprevalence rate or antibody concentrations, in contrast to age and comorbidities. The antibody response was found to significantly decrease with increasing age. The antibody response was found to be significantly lower in people with chronic comorbidities. The fact that the immune response induced by some other vaccines is affected by comorbid conditions leading to a lower antibody response appears to be true for the inactivated vaccines [13]. The fact that the antibody response is lower in people over 50 years of age and with chronic diseases indicates the need for a third dose in these people.

To conclude, in this study, early results (step 1) of our project indicated a high seroconversion rate for anti-S-RBD (98.2%), total anti-spike/anti-nucleocapsid-IgG (92.9%) antibodies, and 24–28% incidence of non-serious adverse events. Our results are expected to help in structuring vaccination strategies, including different age groups and among people who had the disease. A relatively lower antibody response in the elderly, in contrast to a relatively higher one in people who had natural COVID-19, can be evaluated as a need for a third dose in people lacking antibody response after the second dose or in the elderly.

## Supplementary Information



## Figures and Tables

**Table 1:** Validity results of the test MAGLUMI 2019-nCoV IgM/IgG (Target: Spike and Nucleocapsid)^1^

Antibody	Performance Measure	Estimate of Performance(95%CI)
IgM	Sensitivity (PPA)	77.5% (69.9%–83.6%)
IgM	Specificity (NPA)	99.6% (97.5%–99.9%)
IgG	Sensitivity (PPA)	100.0% (97.4%–100.0%)
IgG	Specificity (NPA)	99.1% (96.8%–99.8%)
Combined	Sensitivity (PPA)	100% (97.4%–100.0%)
Combined	Specificity (NPA)	98.7% (96.2%–99.5%)
Combined	PPV at prevalence=5%	79.9% (57.2%–92.1%)
Combined	NPV at prevalence=5%	100.0% (99.9%–100.0%)

PPA: Positive percent agreement, NPA: Negative percent agreement, PPV: Positive predictive value, NPV: Negative predictive value, CI: Confidence interval.

1 Food and Drug Administration (2021).

EUA Authorized Serology Test Performance. In: Coronavirus Disease 2019 (COVID-19) Emergency Use Authorizations for Medical Devices [online]. Web site https://www.fda.gov/medical-devices/coronavirus-disease-2019-covid-19-emergency-use-authorizations-medical-devices/eua-authorized-serology-test-performance [29 March 2021].

**Table 2:** Sociodemographic characteristics and COVID-19 history of the participants (Çukurova Univ., Adana-Turkey, 2021).

Characteristics	n (%)
Total	282 (100.0)
**Sex (male/female)**	
Male	123 (43.6)
Female	159 (56.4)
**Age**	
20–29	69 (24.5)
30–39	83 (29.4)
40–49	75 (26.6)
50 and older	55 (19.5)
All group age (X±S.D.), min-max, median (1st–3rd quartile)	39.06 ± 10.65, 21–65, 34 (30–47)
**Working position**	
Doctor	80 (28.4)
Nurse	62 (22.0)
Others*	140 (49.6)
**Chronic disease presence**	**68 (24.2)**
**Natural COVID-19 diagnosed pre-vaccination**	**45 (16.0)**
**Pre-vaccination infection time (X±S.D.), min-max (days), median (1st-3rd quartile)**	**111.27±77.97, 29–330, 90 (60–150)**
0–90 days	26 (57.8)
91 days and over	19 (42.2)
**Severity of pre-vaccination COVID-19 disease**	
Mild	40 (88.9)
Moderate	5 (11.1)

* The group “Others” include emergency medical technicians, biologists, environmental engineers, pharmacists, electricians, EMG technicians, security staff, nursing staff, occupational health and safety experts, chemists, laborants, officers, audiologists, cleaning staff, psychologists, radiology technicians, secretaries, drivers, and mechanists.

**Table 3:** Seroconversion rates at the end of the first month after the second dose of vaccination with the inactivated vaccine by some characteristics of the participants (Çukurova Univ., Adana-Turkey, 2021).

		Total Anti-spike and Anti-nucleocapsid Antibodies						Anti-SARS-CoV-2 S-RBD		
		IgG			IgM			IgG		
Characteristics		Non-reactive*	Reactive**	p	Non-reactive*	Reactive**	p	Non-reactive*	Reactive**	p
	**Total**	20 (7.1)	262 (92.9)		239 (84.8)	43 (15.2)		5 (1.8)	277 (98.2)	
**COVID-19 history**	**Sex**									
Yes	Male	-	17(100.0)	-	13 (76.5)	4(23.5)	0.468	-	17(100.0)	-
	Female	-	28(100.0)		25(89.3)	3(10.7)		-	28(100.0)	
No	Male	10(9.4)	96(90.6)	0.794	85(80.2)	21(19.8)	0.109	3(2.8)	103(97.2)	0.659
	Female	10(7.6)	121(92.4)		116(88.5)	15(11.5)		2(1.5)	129(98.5)	
	**Age**									
Yes	20–29	-	12(100.0)	-	11(91.7)	1(8.3)	0.439	-	12(100.0)	-
	30–39	-	20(100.0)		15(75.0)	5(25.0)		-	20(100.0)	
	40–49	-	9(100.0)		8(88.9)	1(11.1)		-	9(100.0)	
	50 and above	-	4(100.0)		4(100.0)	0(0.0)		-	4(100.0)	
No	20–29	3(5.3)	54(94.7)	0.540	52(91.2)	5(8.8)	0.347	2(3.5)	55(96.5)	0.456
	30–39	4(6.3)	59(93.7)		51(81.0)	12(19.0)		0(0)	63(100.0)	
	40–49	7(10.6)	59(89.4)		54(81.8)	12(18.2)		1(1.5)	65(98.5)	
	50 and above	6(11.8)	45(88.2)		4(86.3)	7(13.7)		2(3.9)	49(96.1)	
	**Any chronic disease**									
Yes	Yes	-	10(100.0)	-	8(80.0)	2(20.0)	1.000	-	10(100.0)	-
	No	-	35(100.0)		30(85.7)	5(14.3)		-	35(100.0)	
No	Yes	7(12.1)	51(87.9)	0.280	51(87.9)	7(12.1)	0.571	3(5.2)	55(94.8)	0.097
	No	13(7.3)	165(92.7)		149(83.7)	29(16.3)		2(1.1)	176(98.9)	

Chi-square test (Participants with COVID-19 history were excluded in the analyses of sex, age, presence of any chronic disease).

* Number and percent of non-reactive (<1.00 AU/mL), and

** Reactive (≥1.00 AU/mL) participants.

**Table 4:** Antibody concentrations at the end of the first month after the second dose of vaccination with the inactivated vaccine by some characteristics of the participants (Çukurova Univ., Adana-Turkey, 2021).

	Total Anti-spike and Anti-nucleocapsid						SARS-CoV-2 S-RBD		
	IgG (AU/mL)			IgM (AU/mL)			IgG (AU/mL)		
	X±S.D.	Median (Min-Max)	p	X±S.D.	Median (Min-Max)	p	X±S.D.	Median (Min-Max)	p
**Total**	37.33 ± 50.57	19.80 (0.02-367.70)		0.92 ± 1.40	0.48 (0.08–9.02)		44.99 ± 44.66	29.62 (0.10–287.50)	
**Characteristics**									
**COVID-19 history**									
Yes (n:45)	89.74 ± 66.24	75.27 (10.16–292.40)	**<0.001** *	0.73 ± 0.87	0.47 (0.13–3.74)	0.615*	58.31 ± 57.90	38.99 (7.18–287.50)	**0.033** *
No (n:237)	27.37 ± 40.05	14.03 (0.02–367.70)		0.96 ± 1.48	0.48 (0.08–9.02)		42.46 ± 41.09	27.90 (0.10–204.20)	
**Sex**									
Male (n:106)	29.14 ± 46.13	14.39 (0.37–367.70)	0.788*	1.12 ± 1.69	0.52 (0.08–9.02)	0.138*	43.02 ± 42.41	26.84 (0.10–146.20)	0.850*
Female (n:131)	25.95 ± 34.48	14.03 (0.02–198.01)		0.82 ± 1.28	0.43 (0.12–7.59)		42.02 ± 40.15	29.23 (0.47–204.20)	
**Age**									
20–29 (n:57)	33.47 ± 34.07	26.26a,b (0.54–135.60)	**0.038** **	0.92 ± 1.62	0.48 (0.14–7.74)	0.938**	47.09 ± 43.25	33.54^a^ (0.33–161.60)	**0.008** **
30–39 (n:63)	26.01 ± 28.31	18.30 (0.48–143.50)		1.00 ± 1.41	0.46 (0.12–7.11)		51.18 ± 45.96	30.07^b^ (1.96–204.20)	
40–49 (n:66)	22.22 ± 35.04	9.40^b^ (0.36–168.90)		1.00 ± 1.46	0.49 (0.12–7.59)		41.69 ± 39.36	23.45 (0.60–129.70)	
50 and above (n:51)	28.91 ± 60.00	6.14^a^ (0.20–367.70)		0.89 ± 1.48	0.49 (0.08–9.02)		27.52 ± 29.87	14.98a,b (0.10–14.30)	
**Any chronic disease**									
Yes (n:58)	32.32 ± 59.96	7.78 (0.20–367.70)	0.444*	0.88 ± 1.45	0.52 (0.08–9.02)	0.827*	38.07 ± 44.11	17.34 (0.10–204.20)	**0.044** *
No (n:178)	25.76 ± 31.15	16.00 (0.36–168.00)		0.98 ± 1.50	0.47 (0.12–7.74)		43.94 ± 0.20	29.42 (0.33–161.60)	

* Mann–Whitney U test,

** Kruskal–Wallis test (Participants with COVID-19 history were excluded in the analyses of sex, age, presence of any chronic disease).

a,b The cells with the same symbol are statistically significantly different as revealed by post-hoc analyses.

**Table 5:** Incidences of adverse events after the first and second doses of vaccination with the inactivated vaccine (Çukurova Univ., Adana-Turkey, 2021).

Adverse events (n=282)	After 1st dose n(%)	After 2nd dose n(%)
**Total**	**84 (29.8)**	**68 (24.1)**
Pain at the injection site	45 (16.0)	44 (15.6)
Weakness	30 (10.6)	29 (10.3)
Fatigue	28 (9.9)	23 (8.2)
Headache	22 (7.8)	21 (7.4)
Muscle pain	12 (4.3)	5 (1.8)
Back pain	11 (3.9)	2 (0.7)
Joint pain	9 (3.2)	6 (2.1)
Dizziness	8 (2.8)	5 (1.8)
Numbness in the vaccinated arm	7 (2.5)	8 (2.8)
Vertigo	7 (2.5)	3 (1.1)
Shake	4 (1.4)	2 (0.7)
Increased blood pressure	4 (1.4)	-
Nausea	3 (1.1)	5 (1.8)
Loss of taste	3 (1.1)	2 (0.7)
Chest pain	3 (1.1)	2 (0.7)
Loss of appetite	3 (1.1)	2 (0.7)
Sore throat	3 (1.1)	1 (0.4)
Swelling at the injection site	2 (0.7)	4 (1.4)
Rash at the injection site	2 (0.7)	3 (1.1)
Diarrhoea	2 (0.7)	2 (0.7)
Fever	2 (0.7)	1 (0.4)
Palpitation	2 (0.7)	1 (0.4)
Cough	2 (0.7)	-
Numbness in the tongue	2 (0.7)	-
Decreased blood pressure	2 (0.7)	-
Others*	6 (2.4)	9 (3.5)

Each adverse event percentage was separately calculated from the total (n=282) as there were multiple adverse events in each single participant

* Others: Stiffness at the injection site, Tinnitus, Abdominal pain, Anosmia, Balance problem, Metallic taste

## Footnotes

1 World Health Organisation (2021). WHO Coronavirus (COVID-19) Dashboard [online]. Web site https://covid19.who.int/ [6 May 2021].

2 Snibe Diagnostic (2020). MAGLUMI SARS-CoV-2 S-RBD IgG (CLIA) [online]. Web site http://multimedilab.com/wp-content/uploads/2021/01/MAGLUMI%C2%AE-SARS-CoV-2-Neutralizing-Antibody-CLIA.pdf [29 March 2021].

3 World Health Organisation (2013). Correlates of vaccine-induced protection: methods and implications. In: Immunization, Vaccines and Biologicals [online]. Web site https://apps.who.int/iris/bitstream/handle/10665/84288/WHO_IVB_13.01_eng.pdf [29 March 2021].

Snibe Diagnostic (2020). MAGLUMI 2019-nCoV IgM/IgG Immunoassay [online]. Web site https://www.fda.gov/media/142233/download [29 March 2021].

5 Snibe Diagnostic (2020). Fact Sheet for Healthcare Providers - MAGLUMI 2019-nCoV IgM/IgG [online]. Web site https://www.fda.gov/media/142231/download [29 March 2021].

6 Food and Drug Administration (2021). EUA Authorized Serology Test Performance. In: Coronavirus Disease 2019 (COVID-19) Emergency Use Authorizations for Medical Devices [online]. Web site https://www.fda.gov/medical-devices/coronavirus-disease-2019-covid-19-emergency-use-authorizations-medical-devices/eua-authorized-serology-test-performance [29 March 2021].

7 Snibe Diagnostic (2020). Fact Sheet for Healthcare Providers - MAGLUMI 2019-nCoV IgM/IgG [online]. Web site https://www.fda.gov/media/142231/download [29 March 2021].

8 Food and Drug Administration (2021). EUA Authorized Serology Test Performance. In: Coronavirus Disease 2019 (COVID-19) Emergency Use Authorizations for Medical Devices [online]. Web site https://www.fda.gov/medical-devices/coronavirus-disease-2019-covid-19-emergency-use-authorizations-medical-devices/eua-authorized-serology-test-performance [29 March 2021].

9 World Health Organization (2021). Draft landscape and tracker of COVID-19 candidate vaccines [online]. Web site https://www.who.int/publications/m/item/draft-landscape-of-covid-19-candidate-vaccines [21 April 2021].

10 Jon Cohen (2020). Why do dozens of diseases wax and wane with the seasons—and will COVID-19? [online]. Web site https://www.sciencemag.org/news/2020/03/why-do-dozens-diseases-wax-and-wane-seasons-and-will-covid-19 [21 April 2021]. doi:10.1126/science.abb7234

11 COVID19 Vaccine Tracker Team (2021). Sinovac:CoronaVac Phase 3 Trial Turkey-NCT04582344 [online]. Web site https://covid19.trackvaccines.org/vaccines/7/#trial-nct04508075-669un6-kepec2020 [6 May 2021].

12 Republic of Turkey Ministry of Health (2021). COVID-19 Vaccination Information Platform [online]. Web site https://covid19asi.saglik.gov.tr/?_Dil=2 [28 August 2021].
