# Supplementary material for: SARS-COV-2 vaccine: first-month results of a six-month follow-up study
Source: Turk J Med Sci. 2021 Oct 17;52(1):21–31. doi: 10.3906/sag-2106-63 (PMC10734823; doi:10.3906/sag-2106-63)
Supplement: Supplementary file 1 [file TURKJMEDSCI-52-1-21-Supplemental-Table.docx]

**Supplement 1. Different researches/phase trials discussed in the study and compared for immunogenicity and adverse events**

| **Study description (reference)** | **Phase of trial/regime/dose** | **Immunogenicity - Seroconversion rate (%)** | **Median (95%CI)** | **Adverse events incidence** |
| --- | --- | --- | --- | --- |
|  |  |  |  |  |
| Our study | Not a phase trial  **Emergency Usage Licence**  **0-28-day cohort** | at day 28 after the 2^nd^ dose |  | 29.8% between 1st and 2^nd^ dose  24.1% at day 28 after the 2nd dose |
|  |  | **Total anti-spike/anti-nucleocapsid antibodies** |  |  |
|  | 3 μg | IgM 15.2% | 0.48 AU/mL (0.08-9.02) |  |
|  | 3 μg | IgG 92.9% | 19.80 AU/mL (0.02-367.70) |  |
|  |  | **Anti-S-RBD antibodies** |  |  |
|  |  | IgG 98.2% | 29.62 AU/mL (0.10-287.5) |  |
| Zhang et al.(8) | **Phase 1 (0-14-day cohort)** | at day 14 after the 2^nd^ dose |  | at day 14 |
|  |  | **Neutralising antibodies** |  |  |
|  | 3 μg | 46% |  | 29% |
|  | 6 μg | 50% |  | 38% |
|  | Placebo | 0% |  | 8% |
| Zhang et al.(8) | **Phase 1 (0-28-day cohort)** | at day 28 after the 2^nd^ dose |  | at day 28 |
|  |  | **Neutralising antibodies** |  |  |
|  | 3 μg | 83% |  | **13%** |
|  | 6 μg | 79% |  | 17% |
|  | Placebo | 4% |  | 13% |
| Zhang et al.(8) |  | at day 14 after the 2^nd^ dose |  | at day 14 |
|  | **Phase 2 (0-14-day cohort)** |  |  |  |
|  |  | **Neutralising antibodies** |  |  |
|  | 3 μg | 92% |  | 33% |
|  | 6 μg | 98% |  | 35% |
|  | Placebo | 3% |  | 22% |
| Zhang et al. (8) | **Phase 2 (0-28-day cohort)** | at day 28 after the 2^nd^ dose |  | at day 28 |
|  |  | **Neutralising antibodies** |  |  |
|  | 3 μg | 97% |  | **19%** |
|  | 6 μg | 100% |  | **19%** |
|  | Placebo | 0% |  | 18% |
| Xia et al.(19) |  | at day 14 after the 3^rd^ dose |  | at day 7 |
|  | **Phase 1(0-28-56-day cohort)** | **Neutralising antibodies** |  |  |
|  | 2.5 μg (low-dose) | 100% | GMT 316 (218-457) | 20.8% |
|  | 5 μg (medium-dose) | 95.8% | GMT 206 (123-343) | 16.7% |
|  | 10 μg (high-dose) | 100.0% | GMT 297 (208-424) | 25.0% |
|  | Placebo | 0% | GMT 5 (5-5) | 12.5% |
|  | **Phase 1 (0-28-56-day cohort)** | **Specific IgG-binding antibody responses** |  |  |
|  | 2.5 μg (low-dose) | 100% | GMT 415 (288-597) |  |
|  | 5 μg (medium-dose) | 100.0% | GMT 349 (258-472) |  |
|  | 10 μg (high-dose) | 100.0% | GMT 311 (229-422) |  |
|  | Placebo | 0% | GMT 5 (10-10) |  |
| Xia et al.(19) |  | at day 14 after the 2^rd^ dose |  | at day 7 |
|  | **Phase 2 (0-14-day cohort)** | **Neutralising antibodies** |  |  |
|  | 5 μg (medium-dose) | 97.6% | GMT 121 (95-154) | 6.0% |
|  | Placebo | 0% | GMT 5 (5-5) | 14.3% |
|  | **Phase 2 (0-14-day cohort)** | **Specific IgG-binding antibody responses** |  |  |
|  | 5 μg (medium-dose) | 85.7% | GMT 74 (56-97) |  |
|  | Placebo | 0% | GMT 10 (10-10) | 12.5% |
| Xia et al.(19) |  | at day 14 after the 2^rd^ dose |  | at day 7 |
|  | **Phase 2 (0-21-day cohort)** | **Neutralising antibodies** |  |  |
|  | 5 μg (medium-dose) | 97.6% and | GMT 247 (176-345) | 19.0% |
|  | Placebo | 0% and | GMT 5 (5-5) | 17.9% |
|  | **Phase 2 (0-21-day cohort)** | **Specific IgG-binding antibody responses** |  |  |
|  | 5 μg (medium-dose) | 100.0% and | GMT 215 (157-296) |  |
|  | Placebo | 0% and | GMT 10 (10-10) |  |
| Wu et al.(20) |  | at day 28 after the 2^nd^ dose |  | at day 28 |
|  | **Phase 1 (0-28-day cohort)** | **Neutralising antibodies** |  |  |
|  | 3 μg | 100.0% | GMT 54.9 (38.6-78.2) | 20% |
|  | 6 μg | 95.7% | GMT 64.4 (41.5-99.7) | 22% |
|  | Placebo | 0% |  | 21% |
| Wu et al.(20) |  | at day 28 after the 2^nd^ dose |  | at day 28 |
|  | **Phase 2 (0-28-day cohort)** |  |  |  |
|  |  | **Neutralising antibodies** |  |  |
|  | 1.5 μg | 90.7% | GMT 23.4(19.4-28.3) | 20% |
|  | 3 μg | 98.0% | GMT 42.2(35.2-50.6) | 20% |
|  | 6 μg | 99.0% | GMT 49.9(42.2-58.9) | 22% |
|  | Placebo | 0% |  | 21% |

CI: Confidence interval

GMT: Geometric mean titres
